# Dark–Light Tandem Catalytic Oxidation of Formaldehyde over SrBi_2_Ta_2_O_9_ Nanosheets

**DOI:** 10.3390/molecules28155691

**Published:** 2023-07-27

**Authors:** Weimin Ma, Qing Liu, Yuhan Lin, Yingxuan Li

**Affiliations:** 1 College of Chemistry and Chemical Engineering, Key Laboratory of Chemical Additives for China National Light Industry, Shaanxi University of Science and Technology, Xi’an 710021, China; 44845@sust.edu.cn; 2 School of Environmental Science and Engineering, Shaanxi University of Science and Technology, Xi’an 710021, China; 18856326575@163.com (Q.L.); linyuhan1006@sust.edu.cn (Y.L.); 3 MIIT Key Laboratory of Critical Materials Technology for New Energy Conversion and Storage, School of Chemistry and Chemical Engineering, Harbin Institute of Technology, Harbin 150001, China

**Keywords:** formaldehyde, SrBi_2_Ta_2_O_9_, photocatalysis, dark–light tandem catalytic mechanism

## Abstract

Formaldehyde (HCHO), as one of the main indoor toxic pollutions, presents a great threat to human health. Hence, it is imperative to efficiently remove HCHO and create a good indoor living environment for people. Herein, a layered perovskite material SrBi_2_Ta_2_O_9_ (SBT), was studied for the first time and exhibited superior photocatalytic efficiency and stability compared to commercial TiO_2_ (P25). Furthermore, a unique dark–light tandem catalytic mechanism was constructed. In the dark reaction stage, HCHO (Lewis base) site was adsorbed on the terminal (Bi_2_O_2_)^2+^ layer (Lewis acid) site of SBT in the form of Lewis acid-base complexation and was gradually oxidized to CO_3_^2−^ intermediate (HCHO → DOM (dioxymethylene) → HCOO^−^ → CO_3_^2−^). Then, in the light reaction stage, CO_3_^2−^ was completely converted into CO_2_ and H_2_O (CO_3_^2−^ → CO_2_). Our study contributes to a thorough comprehension of the photocatalytic oxidation of HCHO and points out its potential for day–night continuous work applications in a natural environment.

## 1. Introduction

With the advancement of society and people’s living standards, improving the air quality of the living environment is becoming more and more essential. Volatile organic compounds (VOCs) are considered the biggest killer of indoor environments. HCHO is a ubiquitous and highly toxic indoor air pollutant that poses a significant threat to human health, such as eye and nose irritation, headaches, leukemia, pulmonary diseases, and even cancers [1,2,3,4,5,6]. Therefore, it is imperative to efficiently degrade indoor HCHO and thus improve air quality, which not only reduces the health threats but also complies with health needs. Up to now, in order to present a better living quality, various strategies have been intensively developed for this aim, including physical adsorption [7], chemisorption [8,9], thermal catalytic oxidation [10,11], and photocatalytic oxidation [3,4,6] to degrade and convert HCHO into nontoxic CO_2_ and H_2_O.

Physical adsorption is a process that gathers and holds formaldehyde molecules without changing their chemical structures. Chemisorption is largely limited by the regeneration of the adsorbents. Thermal catalytic oxidation is significantly limited with higher reaction temperatures, which is high energy-consuming and not suitable for application in a natural environment. In strong contrast, solar radiation is an inexhaustible source of clean energy, which has been widely used in H_2_ production [12,13], degradation of organic pollutants in water [14,15,16], degradation of VOCs in the air [3,17], etc. By photocatalytic oxidation, HCHO can be simultaneously oxidized to CO_2_ and H_2_O by light irradiation at room temperature. What is more, no other toxic and harmful substances were detected [18], which is undoubtedly a fascinating strategy. However, it must be pointed out that diverse photocatalysts for HCHO removal efficiency are still limited to a few traditional semiconductors such as TiO_2_, Pt/TiO_2_, g-C_3_N_4_ and zeolitic imidazolate framework-8 (ZIF-8) [19,20,21,22]. Therefore, efficient photocatalysts for HCHO removal need to be developed urgently.

Layered perovskite materials, an emerging class of photocatalysts with unique crystal structures, have attracted increasing attention due to their high chemical stability, high absorption coefficients, and long carrier–diffusion lengths [12,13,14,15,16]. At present, layered perovskite materials have been found to have excellent photocatalytic performances in H_2_ production from water splitting [13], CO_2_ reduction [23], conversion of NO_x_ in air [24,25], etc., but their applications in HCHO removal are rarely studied. SrBi_2_Ta_2_O_9_ (SBT), formed by an alternating stack of perovskite—like layers [(SrTa_2_O_7_)^2−^] and fluorine-like [(Bi_2_O_2_)^2+^] layers, is a typical Aurivillius phase layered perovskite photocatalyst [5,26]. In a previous report by our group, the application of Pt-loaded SBT in the catalytic oxidation of HCHO was investigated for the first time [5]. However, it should be noted that no light source was introduced during the whole reaction process, which indicated that it was only an ordinary catalytic reaction rather than a photocatalytic reaction.

In this work, we designed and focused on pure SBT without any modification, explored its intrinsic performance for photocatalytic oxidation of HCHO under Xe lamp irradiation at room temperature, and conducted an in-depth and detailed mechanism study to analyze the tandem catalytic reaction, aiming to provide more references for the practical application of layered perovskite materials in the field of photocatalytic HCHO removal.

## 2. Results and Discussion

### 2.1. Catalyst Characterization

**Figure 1:**
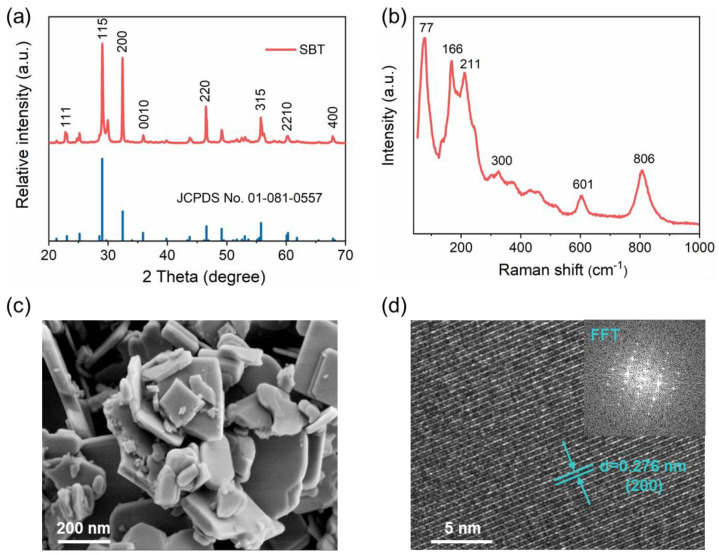
Structural characterization of SBT nanosheet. (**a**) XRD patterns; (**b**) Raman spectra; (**c**) SEM and (**d**) TEM image.

We fabricated an SBT nanosheet photocatalyst by the molten salt method [5]. The crystallographic structure of the as-synthesized SBT sample was confirmed by X-ray diffraction (XRD) measurements. As shown in Figure 1a, the XRD pattern of the SBT sample matched well with the standard card (JCPDS, 01–081–0557) [27], indicating that pure-phase SBT with good crystallinity was successfully synthesized. In addition, Raman analysis was performed to further assess the structure of SBT. As illustrated in Figure 1b, the peak around 806 cm^−1^ belongs to the SBT crystallization degree [28]. The three characteristic peaks in the range of 200–300 cm^−1^ corresponded to O–Ta–O bending vibration, and the band near 600 cm^−1^ assigned to a Ta–O–Ta vibration. These two bands are similar to those found in other perovskite systems, such as KNbO_3_ [28]. Thus, we could conclude that the SBT nanosheet was successfully fabricated.

As shown in Figure 1c, the scanning electron microscopy (SEM) of SBT exhibited a stacked nanosheet structure with a size of 0.1–0.2 μm. Moreover, the high-resolution TEM (HRTEM) image of SBT displayed one set of the perpendicular crystal lattice with clear fringe *d*-spacing of 0.276 nm (Figure 1d), which correspond to the characteristic interlayer distances of the (020) plane in SBT, as well as consistent with the XRD and Raman results. Fast Fourier transform (FFT) pattern analysis of the SBT catalyst further demonstrated the crystal structure, which coincided with the above analysis.

### 2.2. Brunauer–Emmett–Teller (BET) Analysis

**Figure 2:**
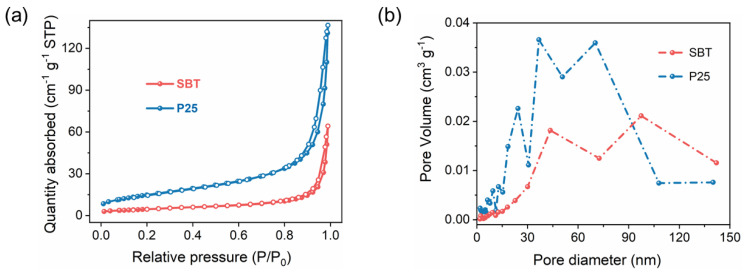
(**a**) Nitrogen adsorption–desorption isotherms and (**b**) the corresponding Barrett–Joyner–Halender (BJH) pore size distribution curves of SBT and P25.

To explore the hierarchical structure of the catalysts, the nitrogen adsorption–desorption isotherms were performed. Specifically, the surface area and pore structure parameters of SBT were determined by Brunauer–Emmett–Teller (BET) method and Barrett–Joiner–Halenda (BJH) method, respectively. P25 was analyzed for comparison. As depicted in Figure 2a, the surface area of SBT (3.22 m^2^/g) was much smaller than those of P25 (54.5 m^2^/g), while the average pore size of SBT (24.9 nm) was larger than that of P25 (16.1 nm). For easy observation, the adsorption capacity of SBT was expanded five times to plot the adsorption isotherm (Figure 2a). The typical H4-type isotherms with hysteresis loops for both SBT and P25 indicated their mesoporous structures [29]. Furthermore, the pore size distribution (Figure 2b) showed a larger proportion of mesoporous (size between 90 and 140 nm) for SBT than P25, which may imply the more favorable transport of reactants and products, as well as higher HCHO adsorption for subsequent photocatalytic activity.

### 2.3. Optical Properties

**Figure 3:**
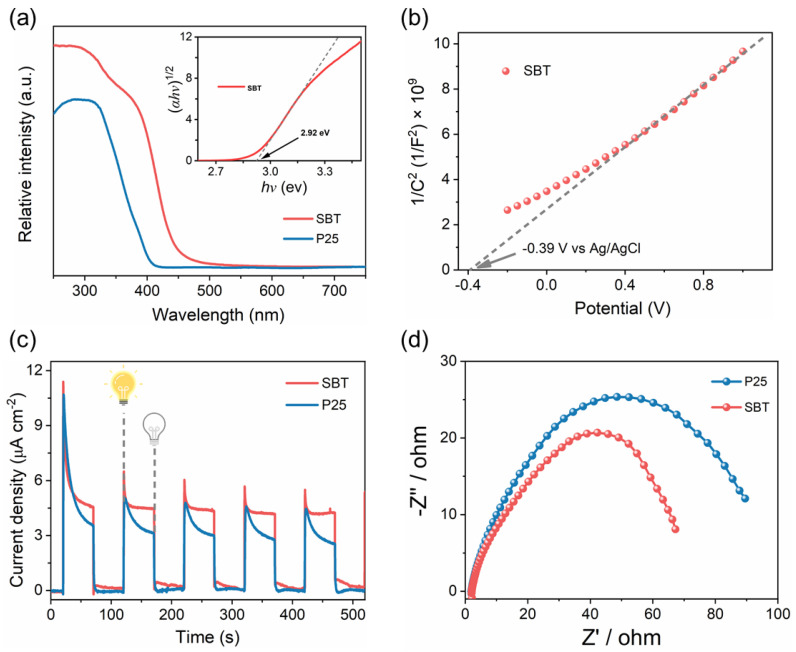
(**a**) UV–Vis diffuse reflectance absorbance spectra (inset is the Tauc plot) of SBT and commercial P25 samples; (**b**) Mott–Schottky plot of SBT; (**c**) transient photocurrent responses, and (**d**) Nyquist plot of impedance data of SBT and P25.

Due to the fact that light absorption is a prerequisite in the photocatalytic HCHO degradation process, the UV–Vis spectrophotometer was carefully investigated to study the light absorption properties of the prepared SBT sample with P25 as the reference (Figure 3a). Compared with P25, the light absorption intensity of SBT was higher, and the absorption edge of SBT shifted to the longer wavelength by 50 nm, expanding an absorption in the visible region. Tauc plot was utilized to evaluate the optical energy band gap of the SBT photocatalyst through the Kubelka–Munk function. According to the previous reports [30,31], in the plot of converted (*αhν*)^r^ versus *hν*, the *r* = 1/2. As shown in the inset of Figure 3a, the Tauc’s band gap (E_g_) of SBT was calculated to be 2.92 eV.

To study the specific band structure of SBT, electrocatalytic measurement was performed. As shown in Figure 3b, the slope of the Mott–Schottky plot is positive, demonstrating that SBT has the characteristics of an n-type semiconductor. The flat band position (E_fb_) of SBT could be determined by the horizontal intercept of the line, which is −0.39 V vs. Ag/AgCl or −0.19 V vs. NHE. For an n-type semiconductor, its E_fb_ is generally considered to be about 0.1 V negative than its conduction band position (E_CB_) [32]. Therefore, the E_CB_ of SBT is roughly calculated to be −0.29 V. Since the E_g_ of SBT obtained above is 2.92 eV, the valence band position (E_VB_) is determined to be 2.63 V. To compare the charge separation and charge transfer capabilities of SBT and P25, the transient photocurrent response measurements were carried out. The higher current density for SBT than that for P25 in Figure 3c indicated that SBT has a better capacity in the separation and transfer of photoinduced carriers. This also aligned with the electrochemical impedance spectroscopy; the smaller Nyquist arc radius of SBT than P25 (Figure 3d) means that the SBT could efficiently inhibit the recombination of photogenerated electron–hole pairs. Moreover, the photocurrent of SBT attenuated more slowly than that of P25, which indicated that SBT has longer photogenerated electron life and better photo response stability than P25. Therefore, we inferred that SBT has better charge transfer ability and photocatalytic HCHO removal performance.

### 2.4. Photocatalytic Catalytic Performance

**Figure 4:**
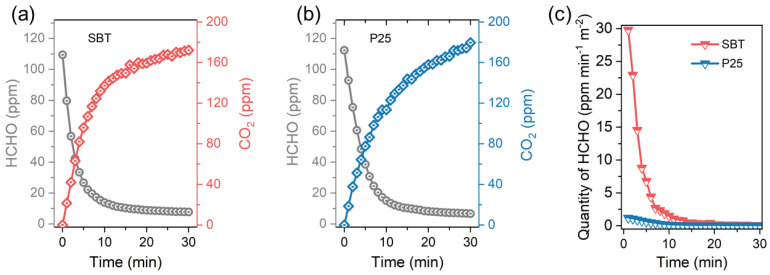
The degradation of HCHO and the formation of CO_2_ over SBT (**a**) and commercial P25 (**b**) catalysts under the illumination of Xe lamp for 30 min; (**c**) the average degradative quantity of HCHO per minute with normalization to BET surface area verse time plots over SBT and P25.

The room temperature activity of SBT for photocatalytic degradation of HCHO was investigated with P25 as the reference in a batch reactor. As shown in Figure 4a,b, after the reactions were carried out under irradiation for 30 min, the HCHO degradation rates of SBT and P25 reached 92.9% and 94.0%, respectively, which indicated that SBT has comparable HCHO degradation performance with P25 at the equal catalyst mass and completely degrade of HCHO molecules. However, for both SBT and P25, the corresponding increase in CO_2_ concentration was much higher than the decrease in HCHO concentration during the first 30 min. This may be caused by the following reasons: (1) On the one hand, during the injection of HCHO, HCHO molecules were primarily adsorbed on the inner wall of the reactor and reached adsorption equilibrium. When HCHO concentration in the reaction system decreased, part of the adsorbed HCHO would be released and then oxidized to CO_2_ at room temperature. (2) On the other hand, during the injection of HCHO, a small amount of HCHO may condense to form solid paraformaldehyde that could not be detected by the gas analyzer. This part of solid paraformaldehyde decomposed into gaseous HCHO molecules again during the reaction, thus becoming an additional carbon source. Therefore, the HCHO oxidation activity was slightly higher than the degradation of HCHO in the initial stage.

To eliminate the difference in HCHO oxidation activity caused by the difference in specific surface area between SBT and P25 catalysts, we normalized the HCHO oxidation activity of the two catalysts according to the specific surface area. Figure 4c shows the two curves of normalized average degradation rates of HCHO against time. Obviously, in the initial stage of the reaction, the HCHO degradation rate over SBT was much higher than that over P25. Especially in the first five minutes when the reactants were sufficient, the rate of photocatalytic degradation of HCHO by SBT was as high as 29.9–4.9 ppm·min^−1^·m^−2^, which was 25.1–11.3 times higher than P25. In a nutshell, the above results demonstrated that the excellent SBT can be used as a substitute catalyst for P25 for photocatalytic HCHO elimination.

**Figure 5:**
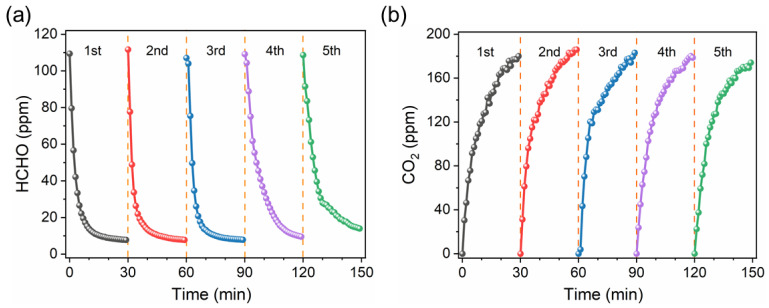
Cycle test for photocatalytic (**a**) degradation of HCHO and (**b**) formation of CO_2_ with SBT sample.

In addition, high stability is another important criterion and major pursuit. To evaluate the photocatalytic HCHO degradation stability of the SBT catalyst, we performed consecutive cycling experiments under the same conditions. As shown in Figure 5a,b, there is a negligible decrease in the level of the HCHO degradation rate by the long-term reaction. Moreover, for each cycle, the amount of CO_2_ production was always positively correlated with the amount of HCHO degradation, which indicated that the SBT catalyst could still completely oxidize HCHO into CO_2_ and H_2_O under a long-time working condition, further demonstratung the high reusability of SBT catalyst and its broad application prospects in the field of photocatalytic HCHO degradation.

### 2.5. Reaction Mechanism

**Figure 6:**
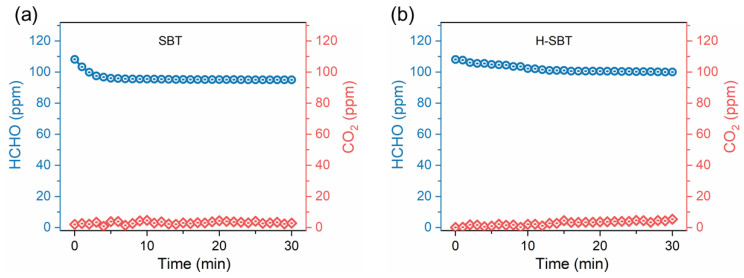
The performance of HCHO adsorption in dark conditions on SBT (**a**) and H-SBT (**b**).

The gas–solid heterogeneous photocatalytic process generally includes two stages: the adsorption stage and the reaction stage. To well elucidate the mechanism of photocatalytic HCHO removal over SBT, a staged experiment was designed. Before the staged experiment, HCHO was injected into the closed system within SBT to determine the adsorption equilibrium time under dark conditions. As shown in Figure 6a, the initial concentration of HCHO in the reactor was 110 ppm, and HCHO concentration gradually decreased with time and remained almost unchanged at 95 ppm after 6 min.

To further explore the influence of the structural components of SBT material on photocatalytic HCHO degradation, the [(Bi_2_O_2_)^2+^] layers of SBT were removed by acid etching to obtain H-SBT material. Then, H-SBT was used in replace of SBT to repeat the HCHO dark adsorption experiment and the dark–light staged experiment under the otherwise identical conditions. The results are shown in Figure 6b. Compared with the SBT, the adsorption equilibrium of HCHO on H-SBT material took much longer (16 min), and the adsorption capacity of HCHO on H-SBT was lower, presenting an HCHO equilibrium concentration of 100 ppm (Figure 6b). This result supports our view that [(Bi_2_O_2_)^2+^] layers in SBT have a crucial effect on the HCHO adsorption process.

In brief, H-SBT was inferior to SBT in photocatalytic HCHO removal capacity, which demonstrated that the high activity of SBT in photocatalytic degradation of HCHO was closely related to [(Bi_2_O_2_)^2+^] layers. It was reported that the HCHO molecule with lone pair electrons could be regarded as Lewis base [33,34], and the SBT with [(Bi_2_O_2_)^2+^] layer can be behaved as Lewis acid [35]. Therefore, the two could be closely combined by acid-base complexation, which greatly promoted the adsorption process of HCHO on SBT and led to the excellent performance of SBT in photocatalytic HCHO removal.

### 2.6. In Situ DRIFT Spectra

**Figure 7:**
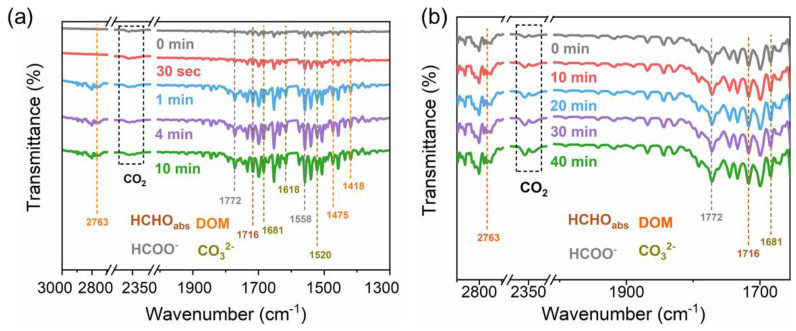
In situ DRIFTS spectra of (**a**) HCHO adsorption reaction in dark condition over SBT sample; (**b**) photocatalytic HCHO degradation reaction over SBT sample (the beginning of spectra is catalyst after exposed to a gas mixture of HCHO, O_2_, and Ar at room temperature for 40 min).

Figure 7a shows the in situ diffuse reflectance infrared Fourier transform spectroscopy of SBT exposed to a certain amount of HCHO, O_2,_ and Ar gas mixture in the dark at different time intervals. The peak at 1716 cm^−1^ indicated that HCHO molecules were adsorbed onto SBT in the form of Lewis acid-base complexation. Peaks at 1418, 1475 and 2763 cm^−1^ corresponded to the vibration of dioxymethylene species (DOM) [36]. Formate species (HCOO^−^) were responsible for the peaks at 1558 and 1772 cm^−1^ [37,38]. Peaks at 1520, 1618, and 1681 cm^−1^ could be ascribed to the carbonate species (CO_3_^2−^) [39], indicating that CO_3_^2−^ species were formed and accumulated during the dark adsorption process of HCHO on SBT. The intensity of the characteristic peaks of adsorbed HCHO and intermediate species (DOM, HCOO^−^, CO_3_^2−^) increased significantly in the initial 1 min and remained stable after 10 min, indicating that the dark adsorption process of HCHO on SBT was very rapid. This was also consistent with the results of the adsorption experiment in Figure 7. In addition, the characteristic peaks at 2335 and 2364 cm^−1^ belonging to CO_2_ molecules showed almost no intensity change during the whole dark adsorption process [40].

All of these suggested that under the dark condition, HCHO molecules adsorbed on the surface of SBT would be rapidly transformed into the intermediate species (DOM, HCOO^−^, CO_3_^2−^) but would not be further converted into CO_2_. Figure 7b gives the in situ DRIFT spectra of the photocatalytic degradation process of HCHO on SBT after the dark adsorption of 30 min. Compared with the dark adsorption process (Figure 7a), no new peak was observed in the photocatalytic degradation process (Figure 7b), implying that the intermediate types generated in the photocatalytic degradation process were consistent with those in the dark adsorption process. In Figure 7b, the peak intensity of DOM, HCOO^−^, and CO_3_^2−^ species exhibited no obvious change with the irradiation time, which indicated that these intermediate species achieved an equilibrium between consumption and generation in the photocatalytic degradation process. However, unlike in Figure 7a, the characteristic peak intensity of CO_2_ molecules in Figure 7b enhanced significantly with the irradiation time. This demonstrated that HCHO adsorbed on the SBT surface would be completely oxidized to CO_2_ with illumination. That is, light was a necessary condition to convert intermediate species such as CO_3_^2−^ into CO_2_. Accordingly, the whole photocatalytic conversion path of HCHO molecules on SBT was supposed to be HCHO → DOM → HCOO^−^ → CO_3_^2−^ → CO_2_.

### 2.7. EPR Measurements

**Figure 8:**
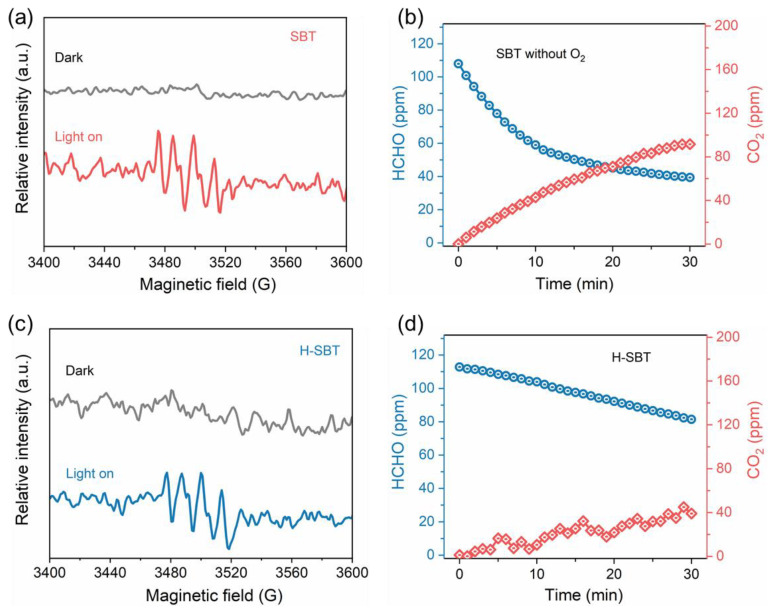
(**a**) EPR spectra for ·O_2_^−^ under dark and light conditions on SBT; (**b**) the performance of photocatalytic HCHO oxidation without O_2_ on SBT; (**c**) EPR spectra for ·O_2_^−^ under dark and light conditions on H-SBT; and (**d**) the performance of photocatalytic HCHO oxidation on H-SBT.

EPR measurements were carried out to investigate the active radical oxygen species in the whole process of photocatalytic HCHO conversion on SBT. Figure 8a displays the EPR spectra of SBT under dark and light conditions. A silent signal under dark conditions and a strong signal ascribed to DMPO −·O_2_^−^ under light conditions were, respectively, detected [41], which implied that photogenerated electrons (e^−^) could combine with O_2_ adsorbed on the SBT surface to form activated superoxide radicals (·O_2_^−^) in the process of photocatalytic HCHO conversion. The poor photocatalytic degradation efficiency of HCHO by SBT in the absence of O_2_ further proved the key role of ·O_2_^−^ in the whole reaction (Figure 8b). Figure 8c shows the EPR spectra of H-SBT under dark and light conditions, and the result was similar to that of SBT. It indicated that the removal of the [(Bi_2_O_2_)^2+^] layers had no obvious effect on the formation of ·O_2_^−^, but the oxidative activity of formaldehyde is greatly weakened (Figure 8d). Hence, these observations demonstrate that the poorer HCHO removal efficiency in the dark–light staged for H-SBT (Figure 6b) than SBT (Figure 6a) should be attributed to the limited formation of the Lewis acid-base complex rather than the ·O_2_^−^ formation.

### 2.8. Tandem Catalytic Mechanism

**Figure 9:**
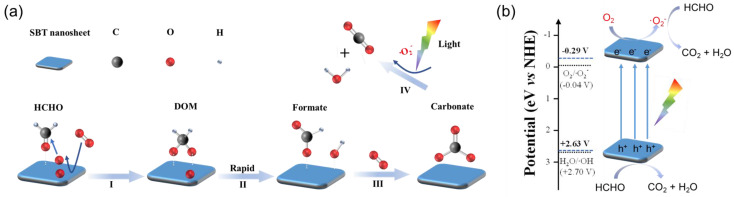
(**a**) Illustration of the tandem adsorption-photodegradation reaction mechanism over SBT nanosheets for HCHO degradation. Specifically, I–IV represents (I) HCHO adsorption and transformed into DOM; (II) DOM rapidly oxidized to the HCOO^−^ species; (III) HCOO^−^ species oxidized into CO_3_^2−^ species; (IV) Under light irradiation, ·O_2_^−^ promotes CO_3_^2−^ convert into CO_2_ and H_2_O. (**b**) Diagram for the band energy levels of SrBi_2_Ta_2_O_9_ and photocatalytic reaction mechanism for HCHO degradation.

Based on the above results, a dark–light tandem catalytic mechanism was proposed for the conversion of HCHO to CO_2_ over SBT. As shown in Figure 9a, under the dark condition, HCHO is adsorbed onto the terminal [(Bi_2_O_2_)^2+^] layer of SBT in the form of a Lewis acid–base complex. Molecular oxygen (O_2_) adsorbed on the surface of SBT decomposes into active oxygen species, which can transform HCHO into DOM. Then, the DOM intermediate is oxidized to the HCOO^−^ species quickly due to its chemical instability. In the presence of sufficient O_2_, HCOO^−^ species will be further oxidized into CO_3_^2−^ species. However, the conversion from CO_3_^2−^ species to CO_2_ is hindered without light. Under light irradiation, the above process of HCHO → DOM → HCOO^−^ → CO_3_^2−^ can also occur, but the difference is that SBT can be excited under irradiation to produce electrons and holes, thus leading to the formation of ·O_2_^−^ through the reaction of photogenerated electrons with oxygen adsorbed on SBT. ·O_2_^−^ can not only promote the process of HCHO → DOM → HCOO^−^ → CO_3_^2−^, but also completely convert CO_3_^2−^ into CO_2_ and H_2_O. Besides, the photogenerated holes (h^+^) with strong oxidation capability can also directly oxidize HCHO to CO_2_ and H_2_O. However, combined with the above energy band results, as illustrated in Figure 9b, the redox potential of H_2_O/·OH is higher than the VB potential of SBT, ·OH production is inhibited at the VB of SBT during the reaction. That is, ·O_2_^−^ and h^+^ are the key active species in the process of photodegradation HCHO on SBT. Based on the above results, we can conclude that SBT firstly transforms HCHO into CO_3_^2−^ intermediate at night and completely converts CO_3_^2−^ intermediate into CO_2_ at daytime.

## 3. Materials and Methods

### 3.1. Catalyst Preparation

SrBi_2_Ta_2_O_9_ (SBT) was synthesized by the molten salt method according to previous reports [5]. Sr(NO_3_)_2_, Bi_2_O_3,_ and Ta_2_O_5_ with a molar ratio of 1:1:1 were used as the raw materials, while KCl and NaCl with a molar ratio of 1:1 were selected as the molten salts. The raw materials and the molten salts were mixed in a molar ratio of 1:1 and grounded in an agate mortar for 30 min. The obtained powder was calcined at 750 °C for 4 h and cooled to room temperature naturally. Then, the resultant was pulverized and rinsed with deionized water to completely remove residual chloride. Finally, the SBT product was obtained after drying at 60 °C overnight. Besides, the H-SBT was obtained by 3 M acid etching 24 h with stirring.

### 3.2. Catalyst Characterization

Powder X-ray diffraction (XRD) patterns were recorded on a Bruker D8 Advance Discover X-ray Diffractometer from the range between 20° to 70° at a scan rate of 0.02° s^−1^. Scanning electron microscope (SEM) was conducted with Hitachi SU8010 Japan. Transmission electron microscopy (TEM) and high-resolution transmission electron microscopy (HRTEM) were conducted with JEOL JEM 2100 Japan. The BET surface areas and the pore size distribution of samples were analyzed by a Micromeritics ASAP 2460 nitrogen adsorption analyzer. The UV–Vis diffuse reflectance spectra (DRS) were obtained by a UV-Vis-NIR spectrophotometer (SHIMADZU UV—2600) with BaSO_4_ as the reflectance standard. Electrochemical experiments were conducted in a 0.5 M Na_2_SO4 solution by a three-electrode system using Chenhua CHI660E electrochemical workstation.

### 3.3. Photocatalytic Tests

The photocatalytic tests for HCHO oxidation were carried out in an airtight reactor (V = 500 mL) at room temperature. First, 0.3 g catalyst powder was uniformly spread on a glass dish (d = 45 mm) in the bottom of the reactor. Then, a certain amount of HCHO was injected into the reactor to ensure that the initial concentration of HCHO in the reactor was 110 ppm. Finally, a 300 W Xe lamp (CEL–PE300L-3A) was used in the top irradiation way to initiate the photocatalytic HCHO oxidation. Concentrations of HCHO, CO_2_ and water vapor were analyzed using an online photoacoustic IR multi–gas monitor (Innova 1412). In the recycling experiments, adsorbed molecules on the catalyst were removed by vacuum treatment at room temperature, and reintroduced HCHO molecules.

### 3.4. Dark–Light Staged Experiment

Dark–light staged experiment included the dark adsorption process of HCHO and the photocatalytic degradation process of the adsorbed HCHO. First, a constant concentration of HCHO was continuously introduced into the reactor containing the SBT sample by flowing 20 *vol*.% O_2_ in Ar over paraformaldehyde placed in a thermostatic water bath. The concentration of HCHO in the reactor was determined as 110 ppm, and the dark adsorption process lasted for 30 min. Then, the introduction of HCHO was stopped. After gaseous HCHO in the reactor was discharged by the O_2_-Ar mixture, the light source was turned on to initiate the photocatalytic degradation reaction of the adsorbed HCHO.

## 4. Conclusions

In summary, the completely photocatalytic degradation of HCHO over pure SBT at room temperature was first investigated, which exhibited the degradation efficiency equivalent to commercial P25. Mechanistic studies showed that the photocatalytic conversion of HCHO to CO_2_ over SBT is a unique dark–light tandem catalytic path: the conversion of HCHO → DOM → HCOO^−^ → CO_3_^2−^ can undergo under dark or light conditions, while the conversion of CO_3_^2−^ to CO_2_ (the complete degradation of HCHO) can only occur under the light irradiation. ·O_2_^−^ generated under light conditions has been proven to be the crucial active species to convert CO_3_^2−^ into CO_2_. Such a dark–light tandem catalytic mechanism enables SBT to work day and night in the practical application environment. Additionally, the stability was proved by consecutive cyclic experiments. In brief, our study firstly demonstrates the superiority of SBT for photocatalytic oxidation HCHO under ambient condition and provide a new prospect for the conversion of air pollutants.

## Data Availability

Not applicable.
